# Fc gamma receptors promote antibody-induced LILRB4 internalization and immune regulation of monocytic AML

**DOI:** 10.1093/abt/tbad025

**Published:** 2023-11-02

**Authors:** Joshua W Morse, Xun Gui, Mi Deng, Ryan Huang, Xiaohua Ye, Peng Zhao, Xuejun Fan, Wei Xiong, Chengcheng Zhang, Ningyan Zhang, Zhiqiang An

**Affiliations:** Texas Therapeutics Institute, Brown Foundation Institute of Molecular Medicine, University of Texas Health Science Center at Houston, 1825 Pressler Street, Houston, TX 77030, USA; Texas Therapeutics Institute, Brown Foundation Institute of Molecular Medicine, University of Texas Health Science Center at Houston, 1825 Pressler Street, Houston, TX 77030, USA; Department of Physiology, University of Texas Southwestern Medical Center, 5323 Harry Hines Boulevard, Dallas, TX 75390, USA; Department of Physiology, University of Texas Southwestern Medical Center, 5323 Harry Hines Boulevard, Dallas, TX 75390, USA; Texas Therapeutics Institute, Brown Foundation Institute of Molecular Medicine, University of Texas Health Science Center at Houston, 1825 Pressler Street, Houston, TX 77030, USA; Texas Therapeutics Institute, Brown Foundation Institute of Molecular Medicine, University of Texas Health Science Center at Houston, 1825 Pressler Street, Houston, TX 77030, USA; Texas Therapeutics Institute, Brown Foundation Institute of Molecular Medicine, University of Texas Health Science Center at Houston, 1825 Pressler Street, Houston, TX 77030, USA; Texas Therapeutics Institute, Brown Foundation Institute of Molecular Medicine, University of Texas Health Science Center at Houston, 1825 Pressler Street, Houston, TX 77030, USA; Department of Physiology, University of Texas Southwestern Medical Center, 5323 Harry Hines Boulevard, Dallas, TX 75390, USA; Texas Therapeutics Institute, Brown Foundation Institute of Molecular Medicine, University of Texas Health Science Center at Houston, 1825 Pressler Street, Houston, TX 77030, USA; Texas Therapeutics Institute, Brown Foundation Institute of Molecular Medicine, University of Texas Health Science Center at Houston, 1825 Pressler Street, Houston, TX 77030, USA

**Keywords:** antigenic modulation, Fc gamma receptor, immunotherapy, LILRB4, monocytic AML

## Abstract

The immune checkpoint leukocyte immunoglobulin-like receptor B4 (LILRB4) is found specifically on the cell surface of acute monocytic leukemia (monocytic AML), an aggressive and common subtype of AML. We have developed a humanized monoclonal IgG_1_ LILRB4-blocking antibody (h128-3), which improved immune regulation but reduced cell surface expression of LILRB4 in monocytic AML models by 40–60%. Interestingly, most of this effect was neutralized by mutation of the Fc region of the antibody (h128-3/N297A), which prevents interaction with Fc gamma receptors (FcγRs). This suggested that there is FcγR-dependent antigenic modulation underlying h128-3’s effects, a mechanism known to alter the function of antibodies targeting B-cell malignancies. We disrupted the Fc-FcγR interaction pharmacologically and with stable CRISPR-Cas9-mediated genetic knockout of FcγRs in monocytic AML cell lines to investigate the role of FcγR-dependent antigenic modulation in the regulation of LILRB4 by h128-3. When FcγRI is inhibited or removed from the surface of monocytic AML cells, h128-3 cannot optimally perform its blocking function, resulting in activation of the LILRB4 inhibitory receptor and leading to a 15–25% decrease in T-cell-mediated cytotoxicity *in vitro*. In the absence of FcγRI, scaffolding by FcγRIIa allows h128-3 to maintain LILRB4-blocking function. Here we define a FcγR-dependent antigenic modulation mechanism underlying the function of an immunoreceptor blocking antibody for the first time in myeloid malignancy. This research will facilitate the development of safe, precision-targeted antibody therapeutics in myeloid malignancies with greater potency and efficacy.

## Teaser

Statement of Significance: Receptor internalization mediated by the LILRB4 neutralizing antibody h128-3 is in part dependent on the IgG_1_-Fc interaction with FcγRs on monocytic AML. FcγR-dependent antigenic modulation is known to profoundly affect the function of antibodies such as rituximab in B-cell malignancies. Here we characterize it for the first time in AML.

## INTRODUCTION

Acute myeloid leukemia (AML) is the most common acute blood and bone marrow cancer in adults and is characterized by rapid growth of abnormal myeloblastic cells that build up in the bone marrow and blood, interfering with normal hematopoiesis [1]. Approximately 10% of AML cases belong to the monocytic AML (M5-AML) subtype, which carries a poor prognosis. NCI SEER data collected from 2013 to 2019 shows 5-year survival for these patients is a dismal 24–29% [2]. There are currently no FDA-approved targeted therapies for monocytic AML.

In previous studies, we reported that leukocyte immunoglobulin-like receptor B4 (LILRB4), a member of the LILRB immunoreceptor family, is a prognostic biomarker for monocytic AML [3–5]. LILRB4 was reported to support tumors through its dual functions as an immune checkpoint that suppresses T-cell proliferation and as a tumor-promoting molecule that enhances tumor migration [3]. Disrupting the interaction of LILRB4 with its functional ligand ApoE by an efficacious humanized antibody h128-3 reverses this T-cell suppression phenotype and blocks AML development [5]. Thus, targeting LILRB4 with antibodies represents a tumor-specific and effective therapeutic strategy for treatment of monocytic AML.

Interestingly, in some monocytic AML patient-derived xenograft (PDX) mice, lower levels of LILRB4 were observed following treatment with h128-3 than with control hIgG [3]. This antigenic modulation could have been caused by simple receptor clustering and subsequent clathrin-mediated endocytosis. However, studies of antibodies targeting receptors on other neoplastic immune cells (CD20 [6–9], CD22 [8], B-cell receptor [8], DR4 [10, 11], DR5 [10, 11] and CD40 [12, 13]) have shown that FcγRs on target cells mediate antigen modulation and affect antibody function. In the clinic, anti-CD20 antibodies demonstrated poor efficacy in B-cell malignancies with high levels of FcγRIIb expression [14, 15]. Thus, the next-generation anti-CD20 antibodies have been engineered to reduce FcγRIIb interactions [16]. More recently, another functional role of Fc-FcγRIIb crosslinking has been reported in the literature. In these cases, FcγRIIb expressed on tissue-infiltrated immune cells serves as a scaffold for enhancing antibody-induced target antigen clustering and cellular effector functions [10–13]. Monocytic AML does not typically express FcγRIIb, but does express FcγRI and FcγRIIa at moderate to high levels [17]. With this as a background, we aimed to investigate the role of FcγRI and FcγRIIa in the h128-3-induced antigenic modulation of LILRB4.

Here we report that the anti-LILRB4 mAb h128-3 induced internalization and degradation of LILRB4 on monocytic AML cells in an FcγRI-dependent manner. We also confirm that the low-affinity FcγRIIa can scaffold h128-3 and internalize the h128-3/LILRB4 complex, particularly in the absence of functional high-affinity FcγRI, which may be saturated by circulating IgG present at a serum concentration of 7–16 mg/ml in the physiologic setting [18]. This work characterizes a novel mechanism of FcγR-mediated antigenic modulation of LILRB4 in monocytic AML and will aid the development of a new generation of therapeutic antibodies optimized to take advantage of this unique biology in myeloid malignancy while maintaining Fc-mediated immune effector functions such as ADCC and ADCP.

## MATERIALS AND METHODS

### Cell lines

HEK293T and HEK293F cells were obtained from Life Technologies (Carlsbad) and maintained in DMEM supplemented with 10% heat-inactivated fetal bovine serum (FBS) (HyClone) and 100 U/ml penicillin and 100 μg/ml streptomycin (Life Technologies). Human monocytic AML cell line THP-1 was obtained from ATCC and maintained in a humidified atmosphere of 5% CO_2_ at 37°C, in R10 [RPMI supplemented with 10% FBS (HyClone) and 100 U/ml penicillin and 100 μg/ml streptomycin (Life Technologies)]. Human monocytic AML cell line Mono-mac-6 was obtained from DSMZ and maintained in a humidified atmosphere of 5% CO_2_ at 37°C, in RPMI supplemented with 10% FBS (HyClone), 1 mM sodium pyruvate, non-essential amino acids (Thermo Fisher), 10 μg/ml human insulin (Sigma) and 100 U/ml penicillin and 100 μg/ml streptomycin (Life Technologies). All cell lines were routinely tested using a mycoplasma-contamination kit (R&D Systems).

### Construction of stable HEK293T overexpression cell lines

HEK293T expressing human LILRB4 with or without FcγRI were generated using lentivirus. Briefly, the receptor cDNA genes (Sino Biological HG16742 and HG10256) were cloned into pCDH-CMV-MCS-IRES-Puro (for LILRB4) or pCDH-CMV-MCS-IRES-Bls (for FcγRI) vectors downstream of the CMV promoter to create the transfer plasmids. The HEK293T cell lines were generated by transducing with packaged lentivirus (generated using the transfer plasmid, pCMV-VSV-G (Addgene 8454), pCMV delta R8.2 (Addgene 12263)). Cells expressing the transgene were selected by 2.5 μg/ml puromycin (Gibco) with or without 5 μg/ml blasticidin (Gibco) until a sufficient number of cells with transgene emerged. For the cells that co-express LILRB4 and FcγRI, the LILRB4-HEK293T stable cell line was first established using lentivirus and selected under 2.5 μg/ml puromycin. Then, the lentivirus particles with FcγRI were used to transduce LILRB4-HEK293T. The double-positive cell lines were selected with 2.5 μg/ml puromycin and 5 μg/ml blasticidin until a sufficient number of cells with transgene emerged. Expression of LILRB4 and FcγR on these stable cell lines was detected by flow cytometry.

### Stable AMoL FcγR-knockout cell line generation with lentiviral CRISPR-Cas9 system

Lentiviral particles were packaged in HEK293T using previously described methods. THP-1 or Mono-mac-6 cells were infected with doxycycline-inducible Cas9-expressing lentivirus (pCW-Cas9, Addgene 50661). After 1 μg/ml puromycin selection for 2 weeks, the surviving cells were infected with sgRNA-expressing lentivirus, produced by the plasmid modified from pSLQ1651 (Addgene 51024) by replacing the Puro-mCherry with Bls or eGFP for selection and flow cytometry purposes. One control sgRNA (control sgRNA 5′- GAACGACTAGTTAGGCGTGTA −3′) and two FcγR targeting sgRNAs (sgRNA-FcγRI 5′- CTGGGAGCAGCTCTACACAG −3′; sgRNA-FcγRIIa 5′- TGCTGAAACTTGAGCCCCCG −3′), which were designed by an online web tool (http://crispr.mit.edu), were cloned into sgRNA plasmids (NT CTRL-eGFP, FcγRI-Bls and FcγRIIA-eGFP). Bls-expressing cells were selected by 5 μg/ml blasticidin for 2 weeks, and GFP-expressing cells were flow-sorted (BD FACSAria II). Cas9 was then induced by 1 μg/ml doxycycline treatment for 1 week. THP-1 wild-type, FcγRI KO, FcγRIIa KO and FcγRI/IIA DKO cells were stained with allophycocyanin (APC)-conjugated antibodies for FcγRI (Biolegend 305013) and FcγRIIa (Sino Biological 10374-MM02-A) and FcγR knockout cell populations (GFP^+^, APC^−^) were negatively selected by FACS (BD FACSAria II).

### Generation of N297A mutated antibody

In our previous study, we constructed the humanized IgG_1_ anti-LILRB4 mAb h128-3 (wild-type) [5]. The variant containing a modified Fc region was made using site-directed mutagenesis PCR. The modification made was an alanine (A) substitution at amino acid N297 of the CH2 region in the h128-3 heavy chain. Mutated heavy-chain and wild-type light-chain constructs were co-transfected into human embryonic kidney freestyle 293 (HEK293F) cells using transfection reagent PEI (Sigma). After 7 days of expression, supernatants were harvested and antibodies were purified by affinity chromatography using protein A resin, as we previously reported (Repligen) [19].

### ELISA binding assay

Corning 96-well EIA/RIA plates were coated for 18 h at 4°C with LILRB4 recombinant proteins (1 μg/ml) and blocked for 2 h at 37°C with 5% non-fat milk. After washing with PBST three times, 100 μl of serially diluted h128-3 antibodies were added and incubated for 45 min at 37°C. Subsequently, the plates were washed with PBST and incubated for 30 min with HRP-conjugated goat anti-human F(ab')_2_ (Jackson ImmunoResearch Laboratories). The immunoreactions were developed with TMB substrates (Sigma) and stopped by the addition of 2 M sulfuric acid before the plate was read at 450 nm.

### Western blot

Cell lysates prepared from cell culture were subjected to SDS-PAGE separation. Gels were transferred to a polyvinylidene fluoride membrane by standard procedures. Membranes were blotted using a goat anti-rabbit Fc-horseradish peroxidase (Sigma) and images were detected with FluorChem M imager (Cell BioSciences) using enhanced chemiluminescence substrate (Denville Scientific). A stalk region linear epitope targeting mAb generated in house (R8, 2 μg/ml), was used to detect LILRB4. Membranes were incubated with antibody for 18 h at 4°C.

### Flow cytometry analysis

For analysis of cell surface receptors, cells were run on the Sartorius iQue3 instrument based on the manufacturer’s instructions. Briefly, 2–5 × 10^5^ cells were dispensed in 100 μl aliquots and blocked with 300 μg/ml hIgG on ice for 1 h. Primary antibodies (5 μg/ml) were then added for 1 h on ice, followed by the addition of labeled secondary detection antibodies as needed. After washing with PBS buffer, the cells were analyzed for fluorescence intensity. Irrelevant rabbit or human IgG was used as negative control.

### LILRB4 internalization assay

Monocytes were seeded in 24-well plates (2 × 10^5^ cells/well, 1 ml) and incubated with antibodies (0.1–10 μg/ml) for 24 h (or different time points) at 37°C. To check LILRB4 internalization, cells were blocked with 300 μg/ml hIgG, Fc fragment (Jackson ImmunoResearch Laboratories) before staining of surface LILRB4 with 5 μg/ml non-competitive rabbit anti-LILRB4 antibody R193 (generated in-house) and performed by FACS. APC-conjugated goat F(ab′)_2_ anti-rabbit F(ab′)_2_ (Jackson ImmunoResearch Laboratories) was diluted 1/200 and used as detection antibody. Internalization was expressed as percentage of surface LILRB4 change treated with anti-LILRB4 antibodies relative to surface LILRB4 treated with irrelevant IgG control:


\begin{align*}\mathrm{LILRB}4\ \mathrm{internalization}\ \left(\%\right) =\frac{\bar{\mathrm x}\ \mathrm{MFI}\ \mathrm{IgG\ -}\ \mathrm{treated}\ \mathrm{group}- \bar{\mathrm x}\ \mathrm{MFI}\ \mathrm{h}128- 3-\mathrm{treated}\ \mathrm{group}} {\bar{\mathrm x}\ \mathrm{MFI}\ \mathrm{IgG}-\mathrm{treated}\ \mathrm{group}} \times 100.\end{align*}

For examination of antibody-induced LILRB4 internalization, a method has previously been reported that detects the internalized antibody using a pH-dependent fluorescence probe. The probe enables maximum fluorescence signals of antibody under intracellular environment [20]. The internalization of anti-LILRB4 antibody was detected using this method. Briefly, antibodies were conjugated with pHAb Amine Reactive dyes (Promega) and then diluted with cell culture media. Monocytic AML cells were seeded into a 24-well plate (4 × 10^5^ cells/well). A total of 100 μl of medium containing different concentrations of pHAb-conjugated antibodies were added into each well. After incubation at 37°C for 18 h, the internalization of anti-LILRB4 antibodies was measured by flow cytometry of fluorescence in the far-red emission channel following the manufacturer’s instructions. The contribution of *cis* and *trans* interactions to internalization of anti-LILRB4 antibody was also detected using this method. In brief, pHAb-conjugated anti-LILRB4 or irrelevant hIgG antibodies (10 μg/ml) were incubated with donor monocytes (GFP^+^) for 1 h at RT. Acceptor monocytes (GFP^−^) were then added 1:1, and co-cultured cells were incubated at 37°C for various time points. Fluorescence of pHAb-conjugated anti-LILRB4 and irrelevant hIgG antibodies by donor or acceptor monocytes was measured relative to untreated donor or acceptor monocytes by flow cytometry of gated GFP^+^ or GFP^−^ cells, respectively. For example, internalization in donor cells was expressed as a fold change relative to untreated cells: 


\begin{align*} &\mathrm{Normalized}\ \mathrm{pHAb}\ \mathrm{in}\mathrm{ternalization}=\frac{\mathrm{MFI}\ \mathrm{of}\ \mathrm{pHAb}\ \mathrm{treated}\ \mathrm{GFP\ +}\ \mathrm{cells}\ \mathrm{in}\ \mathrm{sample}}{\bar{\mathrm x}\ \mathrm{MFI}\ \mathrm{of}\ \mathrm{untreated}\ \mathrm{GFP\ +}\ \mathrm{cells}}. \end{align*}

### Proximity ligation assay

THP-1 and Mono-mac-6 cell lines were seeded in 24-well plates (2 × 10^5^ cells/well, 1 ml), treated with 5 μg/ml irrelevant hIgG or h128-3 and mounted on glass slides. The slides were prepared with cytospin (Hettich ROTOFIX 32A centrifuge, Germany) at 500 RPM for 5 min and fixed with 4% paraformaldehyde (PFA) for 10 min (room temperature). After blocking with Duolink Blocking Solution (Sigma) for 1 h at 37°C, slides were stained with rabbit IgG control R57.4 (generated in-house) or anti-LILRB4 antibody R193 and mouse anti-FcγRI antibody 10.1 (Biolegend) or mouse anti-FcγRIIA antibody 2C3B11B8 (Sino Biological) for 18 h at 4°C. Slides were then stained with Duolink In Situ Orange Starter Kit Mouse/Rabbit (Sigma) according to the manufacturer’s instructions. Slides were stored for 18 h at 4°C before imaging with a Leica TCS SP5 Confocal Microscope. Image processing and analysis was conducted with Leica LAS X software. The PLA colocalization signal (Cy3) intensity mean gray value (MGV) was normalized to nuclear stain (ToPro3) intensity MGV on all samples. Experimental sample PLA colocalization signal intensities were then normalized to IgG control sample PLA colocalization signal intensity (mean of five representative images) by the following normalization formula: 


\begin{align*} \quad\mathrm{Normalized}\ \mathrm{PLA}\ \mathrm{intensity}\ \left(\%\mathrm{MGV}\right) =\frac{\left(\frac{\mathrm{Cy}3\ \mathrm{MGV}\ }{\mathrm{ToPro}3\ \mathrm{MGV}}\right)-\left(\frac{\mathrm{IgG}\ \mathrm{control}\ \mathrm{Cy}3\ \mathrm{MGV}}{\mathrm{IgG}\ \mathrm{control}\ \mathrm{ToPro}3\ \mathrm{MGV}}\right)}{\left(\frac{\mathrm{Cy}3\ \mathrm{MGV}}{\mathrm{ToPro}3\ \mathrm{MGV}}\right)}\times 100. \end{align*}

### LILRB4 blocking assay

THP-1 cell lines were seeded in 6-well plates (1 × 10^7^ cells/well, 2 ml) and serum-starved for 18 h at 37°C to induce cell cycle synchronization. The cells were then incubated with serum-free media supplemented with PBS, hIgG or h128-3 (10 μg/ml) for 1 h at 37°C. ApoE2 (5 μg/ml, Peprotech) and anti-HLA-DR antibody (5 μg/ml, L243, Biolegend) diluted in PBS were plated on non-treated 6-well tissue culture plates for 1 h at 37°C, blocked with 2% BSA/PBS for 30 min at RT and washed twice with sterile PBS at RT. The treated THP-1 cells were then stimulated with bound ApoE2 and anti-HLA-DR antibody for 15 min at 37°C and lysed for 10 min in 1% NP-40 buffer (Alfa Aesar) at 4°C in the presence of protease and phosphatase inhibitors (cOmplete, PhosSTOP). Five percent of the total protein lysate was collected for western blot detection. LILRB4 was immunoprecipitated from the remaining lysed protein for 18 h at 4°C with high-affinity rabbit anti-LILRB4 antibody (R8, 5 μg/ml) using the Dynabeads Protein A Immunoprecipitation Kit (Thermo Fisher). Input protein lysates and immunoprecipitated LILRB4 were subjected to SDS-PAGE separation, proteins were transferred to polyvinylidene fluoride membrane by standard procedures and membranes were blocked with 5% BSA/TBS-T for 30 min at RT then immunoblotted with mouse anti-pTyr antibody 4G10 (1:1000; Cell Signaling) for 18 h at 4°C. Membranes were washed with TBS-T, stained with TidyBlot Western Blot Detection Reagent:HRP (1:400; Bio-Rad) for 1 h at RT; then, images were detected. Membranes were then stripped with mild stripping buffer (Glycine, 0.2 M, pH 2.2) for 15 min at RT, washed with TBS-T and re-blocked with 5% BSA/TBS-T for 30 min at RT. Stripped membranes were then re-probed with rabbit anti-LILRB4 antibody R8 (2 μg/ml) for 1 h at RT and stained with TidyBlot Western Blot Detection Reagent:HRP (1:400; Bio-Rad) for 1 h at RT before image detection. Images were captured with a FluorChem M imager (Cell BioSciences) using enhanced chemiluminescence substrate (Denville Scientific).

### T-cell cytotoxicity assay

CD3^+^ T cells were isolated from healthy donor PBMC by negative selection using the EasySep Human T-Cell Isolation Kit (Stemcell Technologies) and expanded for 48 h at 37°C in ImmunoCult-XF T-Cell Expansion Medium (Stemcell Technologies) enriched with ImmunoCult Human CD3/CD28 T-Cell Activator (25 μl/10^6^ cells/ml, Stemcell Technologies), rIL-7 (10 ng/ml, Peprotech) and rIL-15 (10 ng/ml, Peprotech). GFP^+^ THP-1 NT CTRL and FcγRI KO cells were seeded in Corning 96-Well U-Bottom Microplates (1.25 × 10^4^ cells/well) in normal R10 media or R10 supplemented with isotype control hIgG (20 μg/ml), h128-3 (20 μg/ml) or Staphylococcal enterotoxin B (4 μg/ml, Fisher) for 15 min at 37°C. Expanded T cells were resuspended in R10 and seeded 1:1 v/v in co-culture with the THP-1 cells to achieve final E:T ratios of 1:1, 4:1 or 8:1. Co-cultured cells were incubated for 24 h at 37°C. Then, plates were centrifuged at 1500 RPM for 5 min at 4°C, washed with 2% BSA/PBS by centrifugation at 1500 RPM for 5 min at 4°C, and live/dead-stained with DAPI (0.5 μg/ml, Thermo), and live THP-1 cells (GFP^+^DAPI^−^) were analyzed by FACS (*n* = 6 biologically independent samples). T-cell cytotoxicity of NT CTRL or FcγRI KO cells was expressed as a percentage of GFP^+^DAPI^−^ THP-1 cells remaining after antibody or toxin treatment relative to the average number of GFP^+^DAPI^−^ THP-1 cells remaining in untreated T-cell co-cultures (*n =* 6) of NT CTRL or FcγRI KO cells, respectively:


\begin{align*} \mathrm{Cytotoxicity}\ \left(\%\right) = 100-\left(\frac{\mathrm{live}\ \mathrm{GFP}+\mathrm{DAPI}-\mathrm{cells},\mathrm{antibody}\ \mathrm{or}\ \mathrm{toxin}\ \mathrm{treated}\ }{\bar{\mathrm x}\ \mathrm{number}\ \mathrm{of}\ \mathrm{live}\ \mathrm{GFP}+\mathrm{DAPI}-\mathrm{cells},\mathrm{untreated}}\right)\times 100.\end{align*}

### Statistical analyses

Statistical analyses were performed with Prism 7.0 (GraphPad software). Statistical differences were determined to be significant at *p* < 0.05 using the two-tailed Student *t* test and the two-tailed Mann–Whitney log-rank test. Data were presented as mean ± SEM. In all figures, ^*^ indicates *p* < 0.05, ^*^^*^ indicates *p* < 0.01, ^*^^*^^*^ indicates *p* < 0.001 and ^*^^*^^*^^*^ indicates *p* < 0.0001.

## RESULTS

### Anti-LILRB4 mAb h128-3 induces LILRB4 internalization in monocytic AML cells

**Figure 1:**
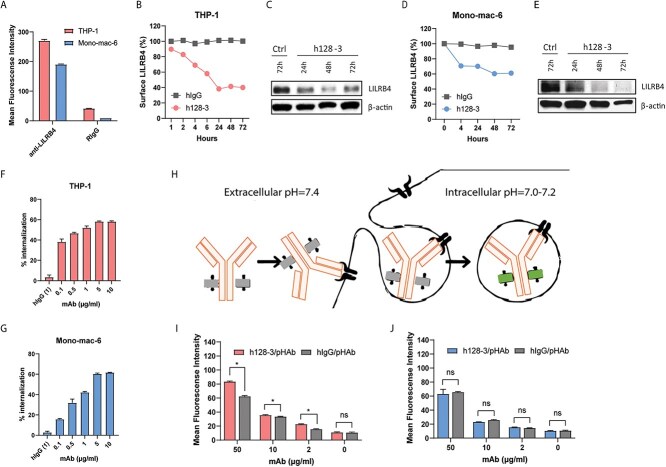
**Antibody h128-3 induces LILRB4 internalization in monocytic AML cells.** (A) Detection of LILRB4 expression on THP-1 and Mono-mac-6 cells by flow cytometry using an anti-LILRB4 rabbit mAb (R193) (*n =* 4). (B, D) Surface LILRB4 downregulation in THP-1 (B) and Mono-mac-6 (D) cells following treatment with h128-3 or control hIgG (*n =* 2). Cells were treated with 10 μg/ml of h128-3 or irrelevant hIgG. At different time points, cells were collected and surface LILRB4 was measured by flow cytometry using anti-LILRB4 rabbit mAb (R193) recognizing a different epitope than that targeted by h128-3. Surface LILRB4 was normalized to hIgG isotype control (100%). (C, E) Total LILRB4 measured by western blot. THP-1 (C) and Mono-mac-6 (E) cells were treated with h128-3, PBS or isotype control hIgG. On hours 24, 48 and 72, cells were collected and LILRB4 was measured by western blot using an anti-LILRB4 rabbit mAb recognizing a linear epitope (R8). (F, G) LILRB4 internalization in THP-1 (F) and Mono-mac-6 (G) cells induced by h128-3 with different concentrations (*n =* 4). Cells were treated with h128-3 and cultured at 37°C for 24 h before measurement of surface LILRB4 by flow cytometry. (H–J) Antibody internalization tested using a pH-dependent dye bound to antibody Fc that fluoresces in acidic intracellular environment (H). h128-3 and isotype control hIgG were conjugated with pH-dependent dye (pHAb) before addition to THP-1 (I) and Mono-mac-6 (J) cells at different concentrations (*n =* 4). Cells were cultured at 37°C for 24 h before fluorescence signal detection by flow cytometry.

We previously generated a panel of anti-LILRB4 mAbs and humanized the lead mAb h128-3, a potential therapeutic candidate for LILRB4-based immunotherapy [4]. In some *in vivo* studies, LILRB4 downregulation was observed [3]. Baseline surface LILRB4 levels in two LILRB4-expressing monocytic AML cell lines (THP-1 and Mono-mac-6) were measured by flow cytometry with the same LILRB4-targeting antibody R193 (Fig. 1A). There were high levels of surface LILRB4 on both cell lines. To confirm potential antibody-mediated LILRB4 downregulation, we carried out a series of *in vitro* studies in these two cell lines. We first treated THP-1 with 10 μg/ml of h128-3 or control hIgG. At different time points, cells were collected and surface LILRB4 was measured by flow cytometry. As shown in Fig. 1B, surface LILRB4 on THP-1 cells was reduced by about 60% following treatment with h128-3 for 24 h. The internalization of h128-3 started immediately following antibody treatment and reached a plateau at 24–48 h. Western blot analysis revealed that total LILRB4 was similarly reduced following treatment with h128-3 (Fig. 1C). We repeated this internalization study in Mono-mac-6 and confirmed that LILRB4 downregulation occurred in this monocytic AML cell line as well **(**Fig. 1D**)**. However, surface LILRB4 on Mono-mac-6 cells was reduced more slowly by h128-3, reaching a maximum of about 40% reduction following treatment with h128-3 for 48 h. Western blot of lysates prepared from these cells revealed that total LILRB4 was similarly reduced (Fig. 1E). We next measured the dose–response of h128-3 for inducing LILRB4 internalization in THP-1 and Mono-mac-6. As shown in Fig. 1F and G, h128-3 has high potency in triggering LILRB4 internalization on THP-1 and Mono-mac-6 cells, respectively. To further confirm LILRB4 internalization, we next labeled h128-3 with a pH-dependent fluorescence probe (pHAb). This probe enables maximum APC-channel fluorescence signals of antibody in the intracellular environment but very weak signal in neutral conditions (Fig. 1H). We treated THP-1 and Mono-mac-6 cells with different concentrations of conjugated antibodies and as shown in Fig. 1I and J, the internalization of h128-3/pHAb is significantly higher than that of isotype control antibody hIgG/pHAb in THP-1 cells but not in Mono-mac-6 cells. This result suggests that the h128-3/LILRB4 complex was internalized in THP-1 cells but h128-3 was not internalized with LILRB4 as a complex in Mono-mac-6 cells.

### Fc engineering disrupts h128-3/LILRB4 internalization in FcγR^high^ monocytic AML

**Figure 2:**
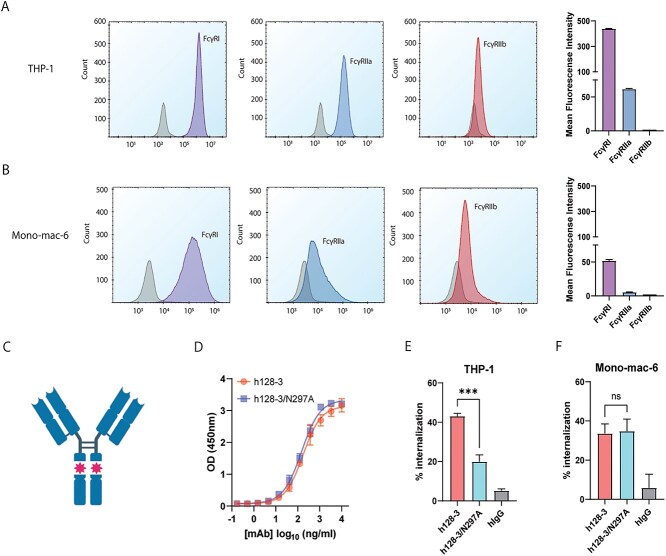
**Prevention of Fc-FcγR crosslinking by Fc engineering decreases LILRB4 internalization in FcγR** ^**high**^ **monocytic AML.** (A, B) The expression level of FcγRs on THP-1 (A) and Mono-mac-6 (B) cells were checked by flow cytometry. All cells except debris were gated on an FSC-A/SSC-A plot. DAPI^−^ cells were then gated and analyzed. Mean fluorescence intensity of FcγRs on THP-1 and Mono-mac-6 was measured by flow cytometry (*n =* 2). (C) Representative graphic of h128-3/N297A Fc-mutant IgG_1_ antibody with significantly decreased affinity for FcγRs. (D) Binding of WT h128-3 or h128-3/N297A to LILRB4 as assessed by ELISA (*n =* 2). (E, F) LILRB4 internalization induced by h128-3, h128-3/N297A or hIgG isotype control in THP-1 (E) or Mono-mac-6 (F) cells (*n =* 4). In brief, 2.5 × 10^5^ cells were treated with 1 μg/ml of antibodies at 37°C for 24 h before measurement of LILRB4 internalization by flow cytometry.

Fc gamma receptors (FcγRs) are known be involved in antigenic modulation on hematopoietic cells [6]. We first measured cell surface levels of the primary FcγRs (FcγRI, FcγRIIa and FcγRIIb) expressed in THP-1 and Mono-mac-6 monocytic cells by flow cytometry. As shown in Fig. 2A, THP-1 cells have relatively high levels of FcγRI expression, moderate levels of FcγRIIa expression and very low levels of FcγRIIb expression. Mono-mac-6 cells have a moderate level of FcγRI expression and low levels of FcγRIIa and FcγRIIb expression (Fig. 2B). The N297A heavy-chain mutation at the h128-3 hinge region is known to significantly reduce the binding affinity between the IgG_1_ Fc and FcγRI, FcγRII, FcγRIII and FcRn due to removal of a glycosylation site on IgG_1_ Fc that promotes FcγR interaction [5, 21–23]. To investigate whether Fc-FcγR scaffolding was involved in LILRB4 internalization, we generated N297A-mutated h128-3 (h128-3/N297A) (Fig. 2C). As expected, h128-3/N297A showed similar levels of binding to LILRB4 as assessed by ELISA (Fig. 2D). h128-3/N297A was then used to test the LILRB4 internalization in FcγR^high^ THP-1 and FcγR^low^ Mono-mac-6 cells for comparison with internalization following treatment with wild-type IgG_1_ h128-3. LILRB4 internalization induced by h128-3/N297A in FcγR^high^ THP-1 cells was significantly lower than the antigenic modulation induced by h128-3 (Fig. 2E). Conversely, in FcγR^low^ Mono-mac-6, h128-3/N297A-induced LILRB4 internalization was not decreased relative to h128-3-induced antigenic modulation (Fig. 2F). These results confirmed the involvement of FcγRs in h128-3/LILRB4 complex internalization in FcγR^high^ THP-1 cells. On the other hand, h128-3-induced antigenic modulation is FcγR-independent in FcγR^low^ Mono-mac-6. A majority of the LILRB4 internalization observed in Mono-mac-6 cells is likely to be mediated by receptor clustering and clathrin-dependent endocytosis.

### FcγRI is involved in LILRB4 internalization induced by h128-3

**Figure 3:**
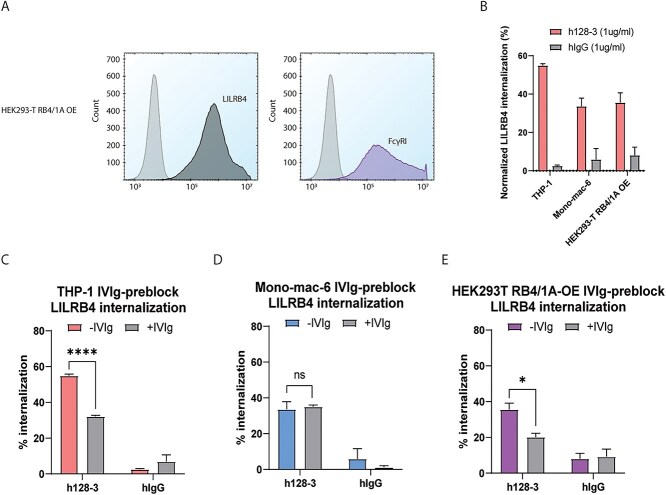
**h128-3-induced LILRB4 internalization in FcγR** ^**high**^ **monocytic AML is promoted by FcγRI crosslinking.** (A) Measurement of the surface expression of LILRB4 and FcγRI on HEK293-T LILRB4/FcγRI OE (HEK293-T RB4/1A OE) cells by flow cytometry. (B) Baseline LILRB4 internalization induced by h128-3 in THP-1, Mono-mac-6 and HEK293T RB4/1A OE cells. Cells were treated with 1 μg/ml of h128-3 or irrelevant hIgG and cultured at 37°C for 24 h before measurement of LILRB4 expression by FACS (*n =* 3). (C–E) h128-3-induced internalization of LILRB4 in THP-1 (C), Mono-mac-6 (D) and HEK293T RB4/1A OE (E) cells blocked with 100-fold irrelevant hIgG (IVIg) prior to h128-3 treatment (*n =* 3). FcγRI on cells was nonspecifically blocked with 1% BSA (-IVIg) or 1% BSA and 100 μg/ml of IVIg (+IVIg) for 30 min at 37°C prior to treatment with 1 μg/ml of isotype control hIgG or h128-3 for 24 h at 37°C. LILRB4 internalization was measured by flow cytometry.

As FcγRI was expressed at moderate to high levels in both monocytic AML cell lines, we wanted to focus on this receptor and study the role of FcγRI independently. To accomplish this, we used HEK293-T cells that do not canonically express LILRB4 or FcγRs and used lentiviral transduction to generate a cell line with dual overexpression of LILRB4 and FcγRI (HEK293-T RB4/1A OE). The overexpression of each non-canonical receptor in HEK293-T was confirmed by flow cytometry (Fig. 3A). We treated each cell line (THP-1, Mono-mac-6 and HEK293-T RB4/1A OE) with h128-3 or irrelevant isotype hIgG (1 μg/ml) for 24 h and found that h128-3-mediated LILRB4 internalization occurs in THP-1 cells, Mono-mac-6 and HEK293-T RB4/1A OE cells (Fig. 3B). To investigate the role of FcγRI in h128-3-mediated LILRB4 internalization, we sought to test whether disrupting the interaction of Fc and FcγR decreases LILRB4 internalization. We pre-incubated the cells with a 100-fold dose of hIgG (IVIg) relative to subsequent h128-3 treatment to block this interaction. FcγRI binds to hIgG_1_ Fc with high affinity (~10^−8^ M) [24]; thus, pre-incubation with a high dose of hIgG will block FcγRI from binding to h128-3 Fc. IVIg pre-incubation prevented about 20% of LILRB4 internalization in FcγR^high^ THP-1 (Fig. 3C) and 15% in FcγR^high^ HEK-T RB4/1A OE (Fig. 3E) but did not prevent LILRB4 internalization in FcγR^low^ Mono-mac-6 (Fig. 3D), confirming that h128-3-mediated LILRB4 internalization in Mono-mac-6 occurs by FcγRI-independent means. These results suggest FcγRI plays a key role in h128-3-induced LILRB4 internalization in FcγR^high^ monocytic AML cells such as THP-1.

### The low-affinity FcγRIIa plays a role in h128-3-induced LILRB4 internalization

FcγRI clearly plays a significant role in the h128-3-induced internalization of LILRB4 on FcγR^high^ monocytic AML THP-1 cells, but we could not rule out the possibility that FcγRIIa can also bind the h128-3 Fc and internalize the h128-3/LILRB4 complex. To characterize the potential role of FcγRIIa in this biological phenomenon, we generated stable FcγRI, FcγRIIa and dual FcγRI/FcγRIIa genetic knockouts in FcγR^high^ THP-1 by CRISPR-Cas9. We specifically generated these knockouts using efficient lentiviral particle delivery of vector DNA encoding doxycycline-inducible Cas9 and FcγR-targeting sgRNAs. We used a vector with a non-targeting sgRNA (NT CTRL) to control for off-target effects of CRISPR-Cas9. Cas9 was doxycycline-induced to perform double-stranded breaks on genes specifically encoding FcγRI and/or FcγRIIa, and knockout cells were negatively selected by FACS (Fig. 4A–C). In FcγR^high^ THP-1, h128-3-induced LILRB4 internalization upon FcγRI knockout at 24 h was significantly decreased relative to internalization in non-targeting control (NT CTRL) cells as expected (Fig. 4D). Interestingly, the h128-3-induced LILRB4 internalization in FcγRIIa KO THP-1 cells was similarly decreased relative to NT CTRL cells (Fig. 4E). In FcγRI/FcγRIIa DKO cells, h128-3-induced LILRB4 internalization was reduced relative to NT CTRL cells by a comparable 25–30% (Fig. 4F). LILRB4 internalization induced by h128-3 in FcγR^high^ THP-1 can thus be mediated by independent contributions from FcγRI or FcγRIIa to achieve its maximal effect.

**Figure 4:**
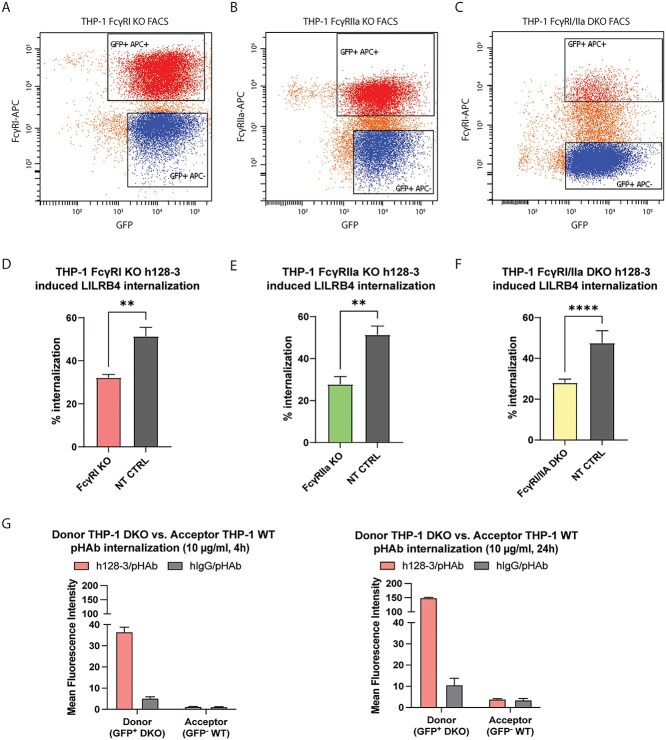
**FcγRIIa contributes to h128-3-induced LILRB4 internalization in FcγR** ^**high**^ **monocytic AML in a time- and FcγRI-dependent manner.** (A–C) FcγRI (A) and FcγRIIa (B) were knocked out in THP-1 cells and FcγRI (C) was knocked out in THP-1 FcγRIIa KO cells by lentiviral particle transduction and positive selection of genes encoding Cas9-PuroR and sgRNA-eGFP or sgRNA-Bls or non-targeting control (NT CTRL) sgRNA-eGFP. Cas9 was doxycycline-induced to perform a double-stranded break of DNA encoding FcγRI or FcγRIIa at the sgRNA-guided sites. Cells were negatively selected by FACS after staining with APC-conjugated anti-FcγRI (10.1, Biolegend) and anti-FcγRIIa (2C3B11B8, Sino) mAbs. (D-F) h128-3-induced LILRB4 internalization in THP-1 NT CTRL cells relative to that in FcγRI KO (*n =* 4) (D) FcγRIIa KO (*n =* 4) (E) or FcγRI/FcγRIIa DKO cells (*n =* 4) (F). In brief, 2.5 × 10^5^ cells were treated with 5 μg/ml of antibodies at 37°C for 24 h before measurement of LILRB4 internalization by flow cytometry. (G) pHAb internalization in THP-1 FcγRI/FcγRIIa DKO (GFP^−^) donor cells or co-cultured THP-1 WT (GFP^+^) acceptor cells. Donor cells were opsonized with 10 μg/ml h128-3/pHAb or hIgG/pHAb and co-cultured 1:1 with acceptor cells for 4 or 24 h at 37°C before pHAb fluorescence in donor and acceptor cells was measured by flow cytometry and normalized to that of untreated co-cultured cells (*n =* 4).

**Figure 4:**
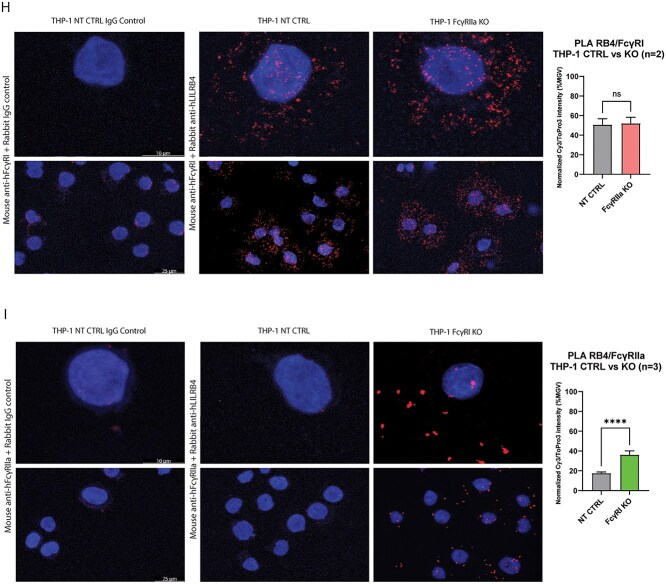
(Continue) (H, I) THP-1 NT CTRL and FcγRIIa KO cells (*n =* 2) (H) or THP-1 NT CTRL and FcγRI KO cells (*n =* 3) (I) were treated with h128-3 (5 μg/ml; 2.5 × 10^5^ cells) for 2 h and fixed on slides. Fixed cells were blocked for 1 h at RT, stained for 18 h at 4°C with mouse anti-hFcγRI/anti-hFcγRIIa and control Rabbit anti-hIgG (all 5 μg/ml) or mouse anti-hFcγRI/anti-hFcγRIIa and rabbit anti-hLILRB4 (all 5 μg/ml) and then stained with the Sigma Duolink Orange (Cy3) PLA kit. Nuclei were stained with ToPro3 and slides were sealed with ProLong Gold Antifade Mountant (ThermoFisher) before imaging with the 63× objective of a Leica TCS SP5 confocal microscope. Five representative images were collected for quantitative analysis of each sample. Image analysis was performed with Leica LAS X software. PLA colocalization signal (Cy3) intensity was normalized to nuclear stain (ToPro3) intensity on all samples. Experimental sample intensities were normalized to mean IgG control sample intensity.

### h128-3/LILRB4 internalization is not driven by FcγRs on neighboring cells

Though studies of rituximab-induced FcγR/CD20 interactions have demonstrated that the antibody–antigen complex can be internalized by FcγR in both *cis* (FcγR on same cell) and *trans* (FcγR on neighboring cell) conformations [6–9], we sought to determine the contribution of *cis* and *trans* FcγRs to h128-3-induced FcγR/LILRB4 interactions. We investigated the *trans* contribution to these interactions empirically using THP-1 FcγRI/FcγRIIa DKO (GFP^−^) donor cells opsonized with 10 μg/ml h128-3/pHAb or hIgG/pHAb and THP-1 WT (GFP^+^) acceptor cells. We incubated cells in co-culture at 37°C, and pHAb fluorescence in the acidic intracellular compartment was measured by flow cytometry at 4 and 24 h. MFI in the far-red channel of GFP^−^ (donor THP-1 DKO) and GFP^+^ (acceptor THP-1 WT) was normalized to that of untreated co-cultured cells (Fig. 4G). Evidently, there are very low levels of internalization of h128-3/pHAb mediated by *trans* FcγRs, while there is significant relative internalization occurring in the THP-1 FcγRI/FcγRIIa DKO cells. This indicates that h128-3-induced LILRB4 internalization is predominantly mediated by *cis* interactions, with minimal contributions from *trans* FcγRs.

### h128-3-induced LILRB4/FcγRIIa colocalization occurs in FcγR^high^ monocytic AML

Given that FcγRI and FcγRIIa can each independently contribute to maximal h128-3-induced LILRB4 internalization in *cis*, we wanted to further clarify the role of each on the scaffolding of h128-3 to target LILRB4 prior to internalization. To resolve this, we utilized an immunohistochemical staining technique known as proximity ligation assay (PLA), which allows the visualization of protein–protein colocalization *in situ* on the surface of cells under high-resolution confocal microscopy. FcγR^high^ THP-1 cells were treated with h128-3 (5 μg/ml) for 2 h, giving LILRB4 and FcγR enough time to colocalize but not enough time for significant h128-3-induced LILRB4 internalization to occur and prevent colocalization signal detection. The cells were then fixed on glass slides, blocked for 1 h at 37°C and stained with primary antibodies for 18 h at 4°C before completing the PLA secondary staining with the Duolink In Situ Orange Starter Kit Mouse/Rabbit the following day. To observe LILRB4/FcγRI colocalization, the h128-3-treated THP-1 NT CTRL or FcγRIIa KO cells were stained with primary antibody combinations of rabbit control IgG and mouse anti-hFcγRI or rabbit anti-hLILRB4 and mouse anti-hFcγRI. As shown in Fig. 4H, there was no PLA colocalization signal detected on the NT CTRL cells stained with rabbit control IgG and mouse anti-hFcγRI, but there was a strong PLA colocalization signal on both the NT CTRL and FcγRIIa KO cells stained with rabbit anti-hLILRB4 and mouse anti-hFcγRI, indicating that rapid h128-3-induced colocalization of LILRB4/FcγRI occurs on the surface of each of these cell populations. Analysis of five representative images each from two independent experiments showed there was no significant difference in normalized LILRB4/FcγRI PLA colocalization signal intensity between NT CTRL and FcγRIIa KO THP-1. To study LILRB4/FcγRIIa colocalization, h128-3-treated THP-1 NT CTRL or FcγRI KO cells were stained with primary antibody combinations of rabbit control IgG and mouse anti-hFcγRIIa or rabbit anti-hLILRB4 and mouse anti-hFcγRIIa. As illustrated in Fig. 4I, there was a very low PLA colocalization signal detected on the NT CTRL cells stained with rabbit control IgG and mouse anti-hFcγRIIa. On the NT CTRL cells stained with rabbit anti-hLILRB4 and mouse anti-hFcγRIIa, there was also very low PLA signal, indicating that h128-3-induced colocalization of LILRB4/FcγRIIa does not occur on the surface of these cells after 2 h. However, when we stained h128-3-treated FcγRI KO THP-1 cells with rabbit anti-hLILRB4 and mouse anti-hFcγRIIa, we found that the PLA signal of LILRB4/FcγRIIa on these cells was relatively high. Analysis of five representative images each from three independent experiments demonstrated a significant increase in normalized LILRB4/FcγRIIa PLA colocalization signal intensity on FcγRI KO THP-1 as compared to NT CTRL. These results indicate that LILRB4 and FcγRI are rapidly colocalized by h128-3 in FcγR^high^ THP-1 prior to h128-3/LILRB4 complex internalization but LILRB4 and FcγRIIa are not. LILRB4 and FcγRIIa colocalization does occur, but it occurs more slowly unless FcγRI is prevented from interacting with LILRB4.

### Interactions between LILRB4 and FcγRI are essential for optimal blocking function of the anti-LILRB4 mAb h128-3 and consequent T-cell-mediated cytotoxicity

**Figure 5:**
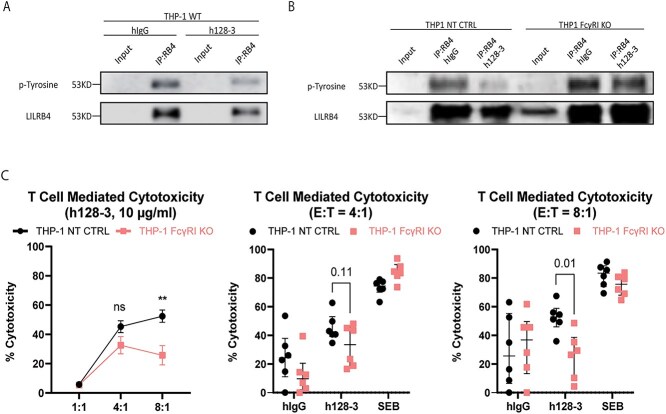
**FcγRI crosslinking enhances the LILRB4 blocking function of h128-3, leading to improved T-cell-mediated cytotoxicity of FcγR** ^**high**^ **monocytic AML.** (A, B) THP-1 WT (A), NT CTRL and FcγRI KO (B) cells (1 × 10^7^) were serum-starved for 18 h and then incubated with PBS, hIgG or h128-3 (10 μg/ml) for 1 h at 37°C. The cells were then seeded on non-treated tissue culture plates with pre-bound ApoE2 (5 μg/ml, Peprotech) and anti-HLA-DR antibody (5 μg/ml, L243, Biolegend) for 15 min at 37°C to stimulate rapid LILRB4 activation before lysis at 4°C in the presence of protease and phosphatase inhibitors. LILRB4 was immunoprecipitated from the lysed cells overnight at 4°C using a high-affinity anti-LILRB4 antibody (R8), and western blot was run on the immunoprecipitated LILRB4. The membrane was probed overnight at 4°C with anti-pTyrosine antibody (4G10, Cell Signaling), stripped with mild stripping buffer and then re-probed with anti-LILRB4 (R8) for 1 h at RT for loading comparison. (C) To determine T-cell-mediated cytotoxicity induced by h128-3, CD3^+^ T cells were first isolated from healthy donor PBMCs by negative selection and expanded for 48 h at 37°C in medium enriched with ImmunoCult Human CD3/CD28 T-Cell Activator (25 μl/10^6^ cells/ml, Stemcell Technologies), rIL-7 and rIL-15 (10 ng/ml, Peprotech). GFP^+^ THP-1 NT CTRL and FcγRI KO cells were then seeded in 96 well U-bottom plates in normal R10 media (untreated) or R10 supplemented with isotype control hIgG (10 μg/ml), h128-3 (10 μg/ml) or Staphylococcus enterotoxin B (2 μg/ml) for 15 min at 37°C. Expanded T cells were then seeded in co-culture with the monocytic AML cells at E:T ratios of 1:1, 4:1 and 8:1 for 24 h at 37°C. The co-cultured cells were washed with 2% BSA/PBS and live/dead-stained with DAPI before analysis by flow cytometry. Live target THP-1 cells (GFP^+^DAPI^−^) were gated and antibody- or toxin-mediated T-cell cytotoxicity of NT CTRL or FcγRI KO THP-1 cells was calculated relative to T-cell cytotoxicity of untreated NT CTRL or FcγRI KO THP-1 cells, respectively.

As we have reported, the LILRB4 blocking antibody h128-3 inhibits monocytic AML development by multiple mechanisms, including reversal of LILRB4-mediated AML tissue infiltration and local T-cell suppression [5]. However, when the Fc region of h128-3 is modified at the N297 residue to reduce FcγR-mediated effector functions, the antibody is not able to optimally induce these phenotypes in FcγR^high^ monocytic AML xenograft mouse models [3, 5]. As others have demonstrated, FcγR on immune cells may serve as a scaffold for enhancing antibody-induced target antigen clustering and effector functions [10–13]. FcγRI binding of h128-3 may similarly guide the antibody to its target, allowing for optimal blocking of LILRB4. To determine if this occurs, we serum-starved FcγR^high^ THP-1 cells to cell-cycle-synchronize them and specifically activated LILRB4 on these cells by receptor clustering using a plate-bound LILRB4 ligand (ApoE, 5 μg/ml). In the presence of LILRB4 ligand and a plate-bound immune stimulant (anti-HLA-DR mAb L243, 5 μg/ml) that can cluster and activate a neighboring ITAM-bearing receptor (HLA-DR), there is tyrosine phosphorylation of LILRB4 that can be detected by western blot of LILRB4 proteins immunoprecipitated from these stimulated THP-1 cells. We first incubated serum-starved THP-1 cells with the LILRB4-blocking antibody h128-3 (10 μg/ml) for 1 h at 37°C to induce h128-3-mediated interactions between LILRB4 and FcγR, before seeding the cells on a plate with pre-bound ApoE and anti-HLA-DR antibody for 15 min at 37°C. The cells were gently lysed in the presence of protease and phosphatase inhibitors at 4°C, and LILRB4 was immunoprecipitated. As expected, western blot detection of tyrosine phosphorylation on LILRB4 immunoprecipitated from the stimulated THP-1 cells pre-treated with h128-3 decreased relative to that of cells pre-treated with isotype control hIgG (Fig. 5A). After confirming that tyrosine phosphorylation on LILRB4 was decreased by h128-3, we sought to determine if FcγRI-scaffolding would improve the blocking function of the antibody. We performed three independent LILRB4 stimulation assays, this time using NT CTRL or FcγRI KO THP-1 cells. Each cell line was serum-starved to induce cell cycle arrest at G0/G1, incubated with h128-3 for 1 h at 37°C and seeded on a plate with pre-bound ApoE and anti-HLA-DR antibody for 15 min at 37°C before gentle cell lysis and immunoprecipitation of LILRB4 protein from each antibody-treated cell population. Each time, the addition of h128-3 to NT CTRL THP-1 cells impeded tyrosine phosphorylation on immunoprecipitated LILRB4 relative to isotype control hIgG as anticipated. However, in FcγRI KO THP-1 cells, h128-3 pre-treatment could not prevent tyrosine phosphorylation relative to the isotype control (Fig. 5B). This indicated that FcγRI scaffolding plays a crucial role in the blocking function of the anti-LILRB4 mAb h128-3 and consequently, its ability to influence T-cell activation and cytotoxicity. To validate this, we isolated CD3^+^ T-cells from healthy donor PBMC and expanded them for 48 h in media enriched with anti-CD3/CD28 agonistic antibodies and IL-7/IL-15 cytokines. As depicted in Fig. 5C**,** we then treated NT CTRL or FcγRI KO THP-1 cells with h128-3 or hIgG isotype control (10 μg/ml) or Staphylococcal enterotoxin B (SEB, 2 μg/ml), a positive control superantigen treatment that induces T-cell hyperactivation and near-total annihilation of neighboring cells [25]. After treatment and T-cell co-culture for 24 h, we assessed T-cell-mediated cytotoxicity of each treatment population relative to that of untreated co-cultured cells. In support of our earlier findings, h128-3 stimulated potent T-cell-mediated cytotoxicity of NT CTRL THP-1 cells in six biologically independent experiments at E:T ratios of 4:1 (35–65%) and 8:1 (35–70%). Though cytotoxicity of FcγRI KO THP-1 co-cultured with expanded CD3^+^ T cells was observed with h128-3 treatment, the observed cytotoxicity was diminished relative to the cytotoxicity of NT CTRL cells at E:T ratios of 4:1 (15–45%, *p* = 0.1054) and 8:1 (5–45%, *p* = 0.0068). This demonstrated that FcγRI on monocytic AML supports h128-3’s ability to influence T-cell activation and cytotoxicity of these malignant cells. FcγRI binding of h128-3 can scaffold the antibody to its LILRB4 target and enhance blocking of its immunosuppressive signaling. Reduction of this inhibitory signal ultimately promotes activation of T cells in the tumor immune microenvironment and T-cell-mediated cytotoxicity of malignant cells.

## DISCUSSION

Despite increased understanding of the underlying biology of AML, the standard intervention of non-targeted cytotoxic chemotherapy followed by consolidative therapy such as bone marrow transplant has not changed in the past 40 years. As many as 70% of patients 65 years or older die of their disease within a year of diagnosis [26, 27]. Moreover, immune checkpoint inhibitor therapeutics, such as those targeting CTLA-4 and PD-1/PD-L1, have not yielded clinical benefits in AML patients with weakened immune function [28, 29]. Recently, our group and others identified LILRB4 as a surface marker for the monocytic AML subtype as it is expressed at significantly higher levels on these cells than on their normal counterparts [3, 30]. Most importantly, LILRB4 expression is inversely correlated with the overall survival of patients with monocytic AML. Binding of ApoE, one of the functional ligands of LILRB4, is coupled with T-cell suppression and tumor infiltration through LILRB4-mediated downstream signaling in AML cells [3]. LILRB4 is thus a promising target for antibody-based therapies for monocytic AML.

We have developed a humanized LILRB4 blocking mAb h128-3, which disrupts the interaction of LILRB4 with ligands including ApoE [5]. h128-3 showed potent activity in blocking the development of monocytic AML in several models, including a xenograft mouse model, a syngeneic immunocompetent mouse model and a PDX mouse model of disseminated AML. We further demonstrated that h128-3 blocks monocytic AML development through reversal of T-cell suppression, inhibition of AML tissue infiltration, ADCC and ADCP [5]. However, in monocytic AML PDX mouse models, we found downregulation of surface LILRB4 following treatment with h128-3 [3]. This phenomenon indicated that h128-3 may induce LILRB4 internalization in an FcγR-mediated fashion. For the receptor CD20 on B-cell lymphoma, FcγRIIb-mediated CD20 internalization with rituximab treatment decreases therapeutic efficacy *in vivo* [7]. FcγR-mediated LILRB4 internalization with h128-3 treatment may represent a different case. As LILRB4 signaling is critical for immune suppression and AML cell tissue infiltration, LILRB4 internalization and degradation induced by h128-3 may block AML cell migration and reverse T-cell suppression permanently. This internalization of LILRB4 induced by h128-3 also suggests that treatment with h128-3-based ADCs may be a useful strategy to deplete monocytic AML cells [31].

Antibody-induced receptor internalization is a commonly occurring biological phenomenon in many cell types that can occur through receptor dimerization and clathrin-mediated endocytosis [32–34]. In this study, we discovered that Fc and FcγR interaction promotes strong h128-3-induced LILRB4 internalization. Disrupting the interaction of Fc and FcγRs by pre-incubation of monocytic AML cells with control hIgG or by CRISPR-Cas9 genetic knockout of FcγRI or FcγRIIa reduced LILRB4 internalization in FcγR^high^ cells such as THP-1. These results are similar to those from the rituximab studies. Rituximab-mediated interactions between CD20 and FcγRIIb on B-cell lymphoma increased the internalization of the rituximab/CD20 immune complex and prevented immune regulation of rituximab-opsonized B-cell lymphoma [6–9]. As monocytic AML is derived from monocytes, it canonically expresses moderate to high levels of FcγRI and FcγRIIa and low levels of FcγRIIb. We confirmed this with flow cytometry, detecting very low levels of FcγRIIb and FcγRIII on monocytic AML cells, which is congruent with other studies [17, 35, 36]. FcγRIII is expressed on NK cells and neutrophils and is responsible for the ADCC effector functions of these cells [37]. We further demonstrated that scaffolding of h128-3 by FcγRI contributes to the internalization of LILRB4. Scaffolding of h128-3 by FcγRIIa also contributes to LILRB4 internalization in the absence of functional high-affinity FcγRI, which may be saturated by circulating IgG in the physiologic setting. Our results suggest that h128-3 can induce interactions between LILRB4 and FcγRI or FcγRIIa on monocytic AML cells, resulting in internalization of the h128-3/LILRB4 complexes and irreversible degradation of LILRB4. However, when THP-1 cells were cultured with h128-3/N297A, a low level of FcγR-independent h128-3-induced LILRB4 internalization was observed. Substitution of glutamine at position 297 to alanine (h128-3/N297A) decreased antibody binding to FcγRs. However, this mutated antibody retains some ability to bind FcγRs [38] and may have retained some FcγR-mediated internalization of LILRB4 relative to isotype control hIgG.

**Figure 6:**
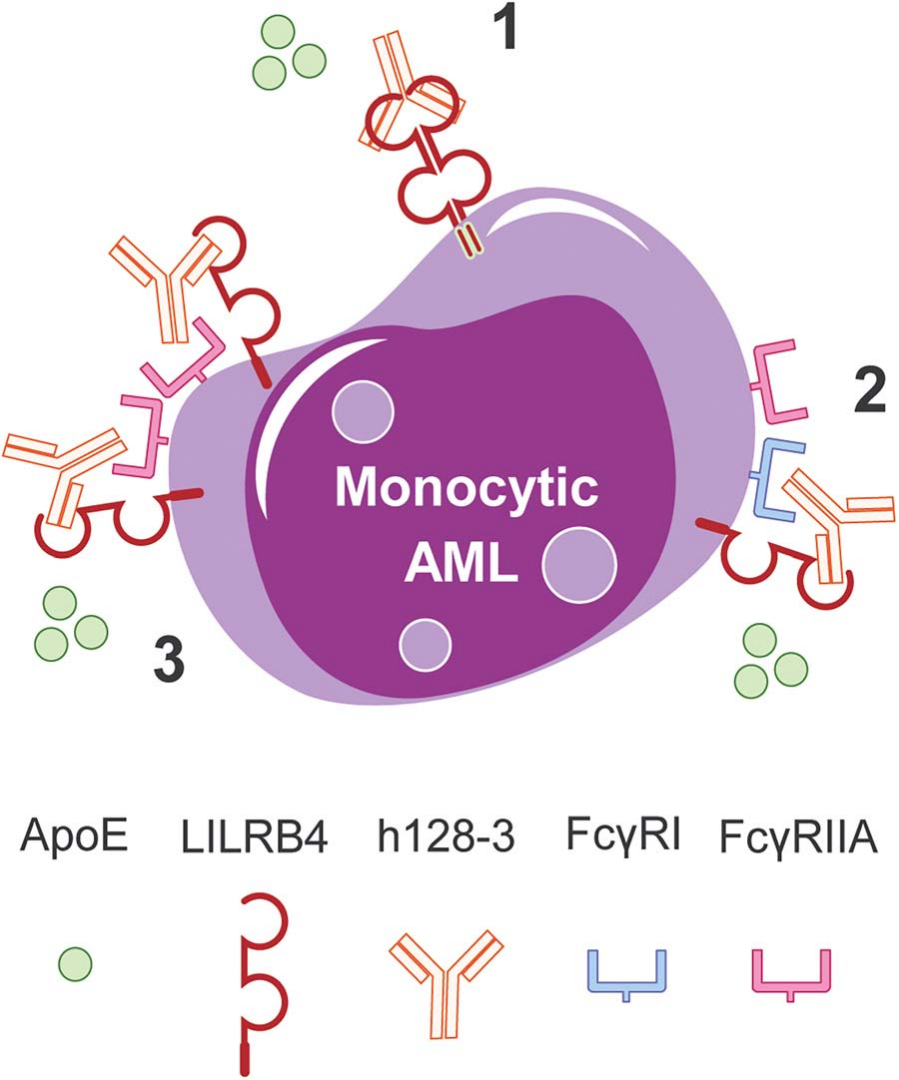
**FcγR-dependent and independent mechanisms of h128-3-induced LILRB4 internalization on monocytic AML.** Depending on the monocytic AML cell surface levels of functional FcγRs, h128-3 binds and internalizes its LILRB4 target receptor on these malignant cells by three potential mechanisms: (1) on all monocytic AML cells, h128-3 crosslinking of LILRB4 receptors triggers clathrin-mediated endocytosis and lysosomal receptor degradation; (2) on FcγR^high^ monocytic AML cells, high-affinity FcγRI scaffolds h128-3 to target LILRB4, leading to internalization and degradation; and (3) on FcγRI^−^FcγRIIA^+^ monocytic AML cells, low-affinity FcγRIIA scaffolds h128-3 by avidity to target LILRB4, prompting its internalization and degradation.

Depending on the FcγR that is scaffolding the h128-3 mAb, the effects on immune regulation may vary. FcγRI and FcγRIIa, the FcγRs that scaffold and internalize the h128-3/LILRB4 complex on monocytic AML cells, both have intracellular immunoreceptor tyrosine-based activating motifs (ITAM) that canonically drive the expression and release of pro-inflammatory cytokines and chemokines (e.g. TNF, IL-1β and IL-8) that alter the effector function, migration and survival of leukocytes [17, 36]. FcγRIIb, the only inhibitory receptor, is expressed on many types of immune cells including B cells, DCs, monocytes, macrophages, mast cells and basophils [39]. The inhibitory FcγRIIb has an intracellular ITIM domain and recruits phosphatases to its ITIM following receptor crosslinking and phosphorylation by Src family kinases. These ITIM-recruited phosphatases, such as SHIP-1 and SHP-2, canonically inhibit immune-activating signals. Crosslinking of FcγRIIb on effector cells by antibody Fc dampens activation of immune cells induced by activating FcγRs through recruitment of these phosphatases [40–42]. However, in some cases, crosslinking of FcγRIIb on immune cells by antibody Fc increases the efficacy of antibodies [10–13]. In one of these cases, enhancing the binding affinity of Fc and FcγRIIb by mutation of some key amino acid residues at the interface increased the therapeutic efficacy of anti-CD40 antibodies *in vivo* [13]. Here, FcγRIIb expressed on tissue-infiltrating immune cells served as a scaffold for enhancing antibody-induced target antigen clustering and cellular effector function. As summarized in Fig. 6, LILRB4-targeting mAb h128-3 takes advantage of similar FcγR-mediated scaffolding effects to bind its target receptor and internalize it. This mechanism leads to improved antagonism of the target inhibitory receptor and activation of effector T cells in the tumor immune microenvironment.

Biologically, our study showcases an example that the functional modification of an inhibitory receptor can be modulated by a local activating receptor. There are several potential directions for future development of LILRB4-based antibody therapeutics. To begin with, we need to balance the antibody-induced LILRB4 internalization on target cells and the effects on immune cells mediated by crosslinking of FcγRs by antibody Fc. For naked therapeutic IgG_1_ mAb development, increasing the interaction of Fc with FcγRI/FcγRIIa on malignant cells or FcγRIII on immune effector cells by generating Fc-engineered mutants or Fc-modified afucosylated variants [43] may be good choices. For development of LILRB4-targeting ADCs [31], we may need to disrupt the paradigm and increase or maintain interactions of Fc with FcγRI/FcγRIIa to obtain significant LILRB4 internalization. In the long run, defining the mechanisms underlying the interaction of therapeutic antibody Fc and FcγRs as we have reported here will help us to develop therapeutics targeting LILRB4 and other immune checkpoints on myeloid malignancies with greater potency and efficacy.

## Data Availability

The datasets used and/or analyzed during the current study are available from the corresponding authors on reasonable request.

## ANIMAL RESEARCH

No animal studies were involved.

## ABBREVIATIONS

ADC: antibody drug conjugate; ADCC: antibody-dependent cell-mediated cytotoxicity; ADCP: antibody-dependent cellular phagocytosis; AML: acute myeloid leukemia; APC: allophycocyanin; ApoE: apolipoprotein E; Cas9: CRISPR-associated protein 9; CRISPR: clustered regularly interspaced short palindromic repeats; DC: dendritic cell; DR4: death receptor 4; DR5: death receptor 5; F(ab’)_2_: bivalent Ag-binding fragment; Fc: fragment crystallizable; FcγRs: Fc gamma receptors; KO: knockout; LILRB4: leukocyte immunoglobulin-like receptor B4; M5-AML: monocytic AML; MFI: mean fluorescence intensity; NT CTRL: non-targeting control; OE: overexpression; PD-L1: programmed death-ligand 1; PD-1: programmed cell death protein 1; PDX: patient-derived xenograft; pHAb: pH-dependent fluorescence probe antibody; PLA: proximity ligation assay; SEB: Staphylococcal enterotoxin B; sgRNA: single-guide RNA

## References

[1] Döhner H, Weisdorf DJ, Bloomfield CD. Acute Myeloid Leukemia. Longo DL (ed.). NEJM 2015; 373: 1136–52. 10.1056/NEJMra1406184 26376137

[2] SEER^*^Explorer: An interactive website for SEER cancer statistics. Surveillance Research Program, National Cancer Institute. Available from https://seer.cancer.gov/statistics-network/explorer/.

[3] Deng, M, Gui, X, Kim, J et al. LILRB4 signalling in leukaemia cells mediates T cell suppression and tumour infiltration. Nature 2018; 562: 605–9. 30333625 10.1038/s41586-018-0615-z PMC6296374

[4] John, S, Chen, H, Deng, M et al. A novel anti-LILRB4 CAR-T cell for the treatment of monocytic AML. Mol Ther 2018; 26: 2487–95. 30131301 10.1016/j.ymthe.2018.08.001 PMC6171100

[5] Gui, X, Deng, M, Song, H et al. Disrupting LILRB4/APOE interaction by an efficacious humanized antibody reverses T-cell suppression and blocks AML development. Cancer Immunol Res 2019; 7: 1244–57. 31213474 10.1158/2326-6066.CIR-19-0036 PMC6677629

[6] Vaughan, AT, Chan, CHT, Klein, C et al. Activatory and inhibitory Fcγ receptors augment rituximab-mediated internalization of CD20 independent of signaling via the cytoplasmic domain. J Biol Chem 2015; 290: 5424–37. 25568316 10.1074/jbc.M114.593806 PMC4342459

[7] Lim, SH, Vaughan, AT, Ashton-Key, M et al. Fc gamma receptor IIb on target B cells promotes rituximab internalization and reduces clinical efficacy. Blood 2011; 118: 2530–40. 21768293 10.1182/blood-2011-01-330357

[8] Vaughan, AT, Iriyama, C, Beers, SA et al. Inhibitory FcγRIIb (CD32b) becomes activated by therapeutic mAb in both cis and trans and drives internalization according to antibody specificity. Blood 2014; 123: 669–77. 24227819 10.1182/blood-2013-04-490821

[9] Dransfield, I . Inhibitory FcγRIIb and CD20 internalization. Blood 2014; 123: 606–7. 24482497 10.1182/blood-2013-12-539874

[10] Li, F, Ravetch, JV. Apoptotic and antitumor activity of death receptor antibodies require inhibitory Fcγ receptor engagement. Proc Natl Acad Sci U S A 2012; 109: 10966–71. 22723355 10.1073/pnas.1208698109 PMC3390832

[11] Wilson, NS, Yang, B, Yang, A et al. An Fcγ receptor-dependent mechanism drives antibody-mediated target-receptor signaling in cancer cells. Cancer Cell 2011; 19: 101–13. 21251615 10.1016/j.ccr.2010.11.012

[12] White, AL, Chan, HTC, Roghanian, A et al. Interaction with FcγRIIB is critical for the agonistic activity of anti-CD40 monoclonal antibody. J Immunol 2011; 187: 1754–63. 21742972 10.4049/jimmunol.1101135

[13] Dahan, R, Barnhart, BC, Li, F et al. Therapeutic activity of agonistic, human anti-CD40 monoclonal antibodies requires selective FcγR engagement. Cancer Cell 2016; 29: 820–31. 27265505 10.1016/j.ccell.2016.05.001 PMC4975533

[14] Lee, CS, Ashton-Key, M, Cogliatti, S et al. Expression of the inhibitory Fc gamma receptor IIB (FCGR2B, CD32B) on follicular lymphoma cells lowers the response rate to rituximab monotherapy (SAKK 35/98). Br J Haematol 2015; 168: 139–59. 25142001 10.1111/bjh.13071

[15] Nowicka, M, Hilton, LK, Ashton-Key, M et al. Prognostic significance of FCGR2B expression for the response of DLBCL patients to rituximab or obinutuzumab treatment. Blood Adv 2021; 5: 2945–57. 34323958 10.1182/bloodadvances.2021004770 PMC8361458

[16] Beers, SA, Chan, CHT, James, S et al. Type II (tositumomab) anti-CD20 monoclonal antibody out performs type I (rituximab-like) reagents in B-cell depletion regardless of complement activation. Blood 2008; 112: 4170–7. 18583569 10.1182/blood-2008-08-172999 PMC2582008

[17] Wang, S, Peng, Y, Wang, R et al. Characterization of neutralizing antibody with prophylactic and therapeutic efficacy against SARS-CoV-2 in rhesus monkeys. Nat Commun 2020 11:1 2020; 11: 1–8. 33188207 10.1038/s41467-020-19568-1 PMC7666115

[18] Whicher, JT . BCR/IFCC reference material for plasma proteins (CRM 470). Clin Biochem 1998; 31: 459–65. 9740967 10.1016/s0009-9120(98)00035-6

[19] Freed, DC, Tang, Q, Tang, A et al. Pentameric complex of viral glycoprotein H is the primary target for potent neutralization by a human cytomegalovirus vaccine. Proc Natl Acad Sci U S A 2013; 110: E4997. 24297878 10.1073/pnas.1316517110 PMC3870741

[20] Nath, N, Godat, B, Zimprich, C et al. Homogeneous plate based antibody internalization assay using pH sensor fluorescent dye. J Immunol Methods 2016; 431: 11–21. 26851520 10.1016/j.jim.2016.02.001

[21] Jefferis, R, Lund, J. Interaction sites on human IgG-Fc for FcγR: current models. Immunol Lett 2002; 82: 57–65. 12008035 10.1016/s0165-2478(02)00019-6

[22] Arnold, JN, Wormald, MR, Sim, RB et al. The impact of glycosylation on the biological function and structure of human immunoglobulins. Annu Rev Immunol 2007; 25: 21–50. 17029568 10.1146/annurev.immunol.25.022106.141702

[23] Shields, RL, Namenuk, AK, Hong, K et al. High resolution mapping of the binding site on human IgG1 for FcγRI, FcγRII, FcγRIII, and FcRn and design of IgG1 variants with improved binding to the FcγR. J Biol Chem 2000; 276: 6591–604. 11096108 10.1074/jbc.M009483200

[24] Bruhns, P, Iannascoli, B, England, P et al. Specificity and affinity of human Fcγ receptors and their polymorphic variants for human IgG subclasses. Blood 2009; 113: 3716–25. 19018092 10.1182/blood-2008-09-179754

[25] Levy, R, Rotfogel, Z, Hillman, D et al. Superantigens hyperinduce inflammatory cytokines by enhancing the B7-2/CD28 costimulatory receptor interaction. Proc Natl Acad Sci U S A 2016; 113: E6437–46. 27708164 10.1073/pnas.1603321113 PMC5081635

[26] Meyers, J, Yu, Y, Kaye, JA et al. Medicare fee-for-service enrollees with primary acute myeloid leukemia: an analysis of treatment patterns, survival, and healthcare resource utilization and costs. Appl Health Econ Health Policy 2013; 11: 275–86. 23677706 10.1007/s40258-013-0032-2

[27] Pulte, D, Redanie, MT, Jansen, L et al. Recent trends in survival of adult patients with acute leukemia: overall improvements, but persistent and partly increasing disparity in survival of patients from minority groups. Haematologica 2013; 98: 222–9. 22929974 10.3324/haematol.2012.063602 PMC3561429

[28] Curran, EK, Godfrey, J, Kline, J. Mechanisms of immune tolerance in leukemia and lymphoma. Trends Immunol 2017; 38: 513–25. 28511816 10.1016/j.it.2017.04.004 PMC6049081

[29] Sehgal, A, Whiteside, TL, Boyiadzis, M. Programmed death-1 checkpoint blockade in acute myeloid leukemia. Expert Opin Biol Ther 2015; 15: 1191–203. 26036819 10.1517/14712598.2015.1051028 PMC4778424

[30] Dobrowolska, H, Gill, KZ, Serban, G et al. Expression of immune inhibitory receptor ILT3 in acute myeloid leukemia with monocytic differentiation. Cytometry B Clin Cytom 2013; 84B: 21–9. 10.1002/cyto.b.21050 23027709

[31] Anami, Y, Deng, M, Gui, X et al. LILRB4-targeting antibody–drug conjugates for the treatment of acute myeloid leukemia. Mol Cancer Ther 2020; 19: 2330–9. 32879051 10.1158/1535-7163.MCT-20-0407 PMC7921214

[32] St. Pierre, CA, Leonard, D, Corvera, S et al. Antibodies to cell surface proteins redirect intracellular trafficking pathways. Exp Mol Pathol 2011; 91: 723–32. 21819978 10.1016/j.yexmp.2011.05.011 PMC3315679

[33] Cheng, J, Liang, M, Carvalho, MF et al. Molecular mechanism of HER2 rapid internalization and redirected trafficking induced by anti-HER2 biparatopic antibody. Antibodies 2020; 9: 49. 32961882 10.3390/antib9030049 PMC7551206

[34] Opaliński, Ł, Sokołowska-Wȩdzina, A, Szczepara, M et al. Antibody-induced dimerization of FGFR1 promotes receptor endocytosis independently of its kinase activity. Sci Rep 2017; 7: 7121. 28769084 10.1038/s41598-017-07479-z PMC5540934

[35] Dugast, AS, Tonelli, A, Berger, CT et al. Decreased Fc receptor expression on innate immune cells is associated with impaired antibody-mediated cellular phagocytic activity in chronically HIV-1 infected individuals. Virology 2011; 415: 160–7. 21565376 10.1016/j.virol.2011.03.012 PMC3112178

[36] Chen, X, Song, X, Li, K et al. FcγR-binding is an important functional attribute for immune checkpoint antibodies in cancer immunotherapy. Front Immunol 2019; 10: 439909. 10.3389/fimmu.2019.00292 PMC6399403 30863404

[37] Junker, F, Gordon, J, Qureshi, O. Fc gamma receptors and their role in antigen uptake, presentation, and T cell activation. Front Immunol 2020; 11: 547589. 10.3389/fimmu.2020.01393 PMC7350606 32719679

[38] Schlothauer, T, Herter, S, Koller, CF et al. Novel human IgG1 and IgG4 Fc-engineered antibodies with completely abolished immune effector functions. Protein Eng Des Sel 2016; 29: 457–66. 27578889 10.1093/protein/gzw040

[39] Smith, KGC, Clatworthy, MR. FcγRIIB in autoimmunity and infection: evolutionary and therapeutic implications. Nat Rev Immunol 2010 10:5 2010; 10: 328–43. 20414206 10.1038/nri2762 PMC4148599

[40] Pearse, RN, Kawabe, T, Bolland, S et al. SHIP recruitment attenuates FcγRIIB-induced B cell apoptosis. Immunity 1999; 10: 753–60. 10403650 10.1016/s1074-7613(00)80074-6

[41] Huang, Z-Y, Hunter, S, Kim, M-K et al. The effect of phosphatases SHP-1 and SHIP-1 on signaling by the ITIM- and ITAM-containing Fcγ receptors FcγRIIB and FcγRIIA. J Leukoc Biol 2003; 73: 823–9. 12773515 10.1189/jlb.0902454

[42] Bournazos, S, Gupta, A, Ravetch, JV. The role of IgG Fc receptors in antibody-dependent enhancement. Nat Rev Immunol 2020 20:10 2020; 20: 633–43. 32782358 10.1038/s41577-020-00410-0 PMC7418887

[43] Pereira, NA, Chan, KF, Lin, PC et al. The “less-is-more” in therapeutic antibodies: Afucosylated anti-cancer antibodies with enhanced antibody-dependent cellular cytotoxicity. MAbs 2018; 10: 693. 29733746 10.1080/19420862.2018.1466767 PMC6150623

